# Specific learning difficulties and intelligence in children with RASopathies. The Grey Matter project

**DOI:** 10.1192/j.eurpsy.2025.1686

**Published:** 2025-08-26

**Authors:** F. Castellano-García, B. Moliner-Castellano, J. Arias-Álvarez, B. Villar-Baptista, L. Solbes-Gálvez, M. Moreno-Ventura, D. M. Peñalver-García, E. Fernández-Jiménez

**Affiliations:** 1 Universidad Cardenal Herrera-CEU, CEU Universities; 2 Private Center Specialized in Neurodevelopmental Disorders, Castellón de la Plana; 3 GOA Center, Oviedo; 4 Consejería de Educación de Madrid, Madrid; 5 CREAS. Espacio de desarrollo y aprendizaje, El Campello, Alicante; 6 University of Murcia, Murcia; 7 Department of Psychiatry, Clinical Psychology and Mental Health, La Paz University Hospital; 8 Hospital La Paz Institute for Health Research (IdiPAZ), Madrid; 9 Faculty of Social Sciences and Communication, Universidad Europea de Madrid, Villaviciosa de Odón, Spain

## Abstract

**Introduction:**

Children with Noonan syndrome, the most prevalent RASopathy, exhibit a characteristic neurocognitive phenotype. Specific difficulties in learning, memory, and executive function are among the most frequently observed manifestations documented in the scientific literature.

**Objectives:**

The present study aimed to measure specific learning difficulties and intelligence in children with RASopathies.

**Methods:**

A total sample of 47 patients (55.3% male) aged 3–16 years (*M* = 8.6, *S.D.* = 3.1) was recruited from various Spanish regions (Murcia, Alicante, Gandía, Valencia, Castellón de la Plana, Tarragona, Cantabria, Asturias, Zaragoza, Madrid, Badajoz, Huelva, Sevilla and Cádiz), within the framework of The Grey Matter project. Most patients were diagnosed with Noonan syndrome, and some had Cardiofaciocutaneous syndrome. The most frequent mutations were PTPN11 (72.3%), RIT1 (6.4%), and unknown mutations in 12.8% of patients. Patients were assessed using the Spanish version of the Wechsler Preschool and Primary Scale of Intelligence, Fourth Edition (WPPSI-IV) or the Wechsler Intelligence Scale for Children, Fifth Edition (WISC-V) according to age; the Spanish Reading Processes Evaluation Battery (PROLEC) according to the appropriate age range (from 6 years); and the Spanish Writing Processes Evaluation Battery (PROESC), from 8 years.

**Results:**

On the one hand, 32.3% presented with severe difficulty in reading words. In turn, 30% of the children showed severe difficulty in reading comprehension of texts when they were read by an examiner, and 29% after reading by themselves. On the other hand, 56% of the children presented severe orthographic difficulty in writing words and 50% presented severe difficulty in written expressions. Finally, regarding intelligence, the mean total IQ of the children was 84.7 points (*S.D.* = 15.1), with better scores on the Verbal Comprehension Index (*M* = 89.4; *S.D.* = 16), Visual Spatial Index (*M* = 87.7; *S.D.* = 13.8), and Fluid Reasoning Index (*M* = 88.6; *S.D.* = 11.9), in contrast with the Working Memory Index (*M* = 84.3; *S.D.* = 13.2), and the Processing Speed Index (*M* = 83.2; *S.D.* = 14.4). In turn, 42.6% of children had low global intelligence scores (IQ ≤ 79).

**Conclusions:**

In conclusion, children with RASopathies, particularly those diagnosed with Noonan syndrome, need educational support to compensate for all these significant academic difficulties.

**Disclosure of Interest:**

None Declared

